# NT-proBNP and the diagnosis of exercise-induced myocardial ischaemia

**Published:** 2008-10

**Authors:** Jacques De Greef, Maryke Funk, William JH Vermaak, Nalini S Perumal, Carlos D Libhaber, Mboyo-DI-Tamba Vangu

**Affiliations:** Department of Chemical Pathology, University of Pretoria, Pretoria; Department of Chemical Pathology, University of Pretoria, Pretoria; Department of Chemical Pathology, University of Pretoria, Pretoria; Department of Nuclear Medicine, Johannesburg and Chris Hani-Baragwanath Hospitals, University of the Witwatersrand, Johannesburg; Department of Nuclear Medicine, Johannesburg and Chris Hani-Baragwanath Hospitals, University of the Witwatersrand, Johannesburg; Department of Nuclear Medicine, Johannesburg and Chris Hani-Baragwanath Hospitals, University of the Witwatersrand, Johannesburg

## Abstract

**Background:**

Amino terminal pro-B-type natriuretic peptide (NT-proBNP) is a sensitive marker of ventricular dysfunction. Exercise causes an increase in the secretion of NT-proBNP, and with myocardial ischaemia the increase is more pronounced. This increase has been found to improve the diagnostic sensitivity of the stress ECG in diagnosing myocardial ischaemia in subjects with normal ventricular function.

**Objective:**

To assess whether the change in NT-proBNP can be used to diagnose effort-induced myocardial ischaemia in an unselected population.

**Methods:**

We enrolled a total of 51 consecutive patients, referred for exercise stress ^99m^Tc-sestamibi SPECT MPI (single-photon emission computed tomography myocardial perfusion imaging) to diagnose inducible myocardial ischaemia. NT-proBNP was determined at rest and 30 minutes after cessation of exercise.

**Results:**

Of the 51 patients, 28 had normal perfusion scans, seven had scans with fixed perfusion defects (previous myocardial infarction with no inducible ischaemia) and 16 had reversible perfusion defects (inducible ischaemia). There was no correlation between ischaemia and resting NT-proBNP, post-stress NT-proBNP or the change in NTproBNP (delta-NT-proBNP).

**Conclusion:**

In an unselected population the change in NT-proBNP cannot be used to diagnose effort-induced myocardial ischaemia.

## Summary

The B-type natriuretic peptides are sensitive markers of ventricular dysfunction and are playing an increasingly important role in the diagnosis, management and prognosis of cardiac disease.^1^-^5^ Exercise has been shown to increase BNP secretion;^6^ in subjects with myocardial ischaemia this elevation is more pronounced and has been found to improve the diagnostic sensitivity of the exercise-stress ECG in a selected population with normal left ventricular ejection fractions.^7^,^8^

Myocardial ischaemia results in ventricular dysfunction and this results in B-type natriuretic peptide (BNP) secretion, however ischaemia *per se* can cause the secretion of BNP.^9^-^12^ This is the rationale behind monitoring the change in amino terminal pro-B-type natriuretic peptide (NT-proBNP) secretion with effort-induced myocardial ischaemia to aid in the diagnosis of myocardial ischaemia.

The B-type natriuretic peptides refer to BNP, the active hormone, and NT-proBNP to the inactive metabolite of the prohormone; both can be measured reliably *in vitro*.^9^ BNP and NTproBNP are secreted in equimolar quantities. BNP has a halflife of 20 minutes and NT-proBNP a half-life of 90 minutes.

These two analytes are metabolised differently and this may play a role in the selection of a particular analyte. However, in comparative studies,^13^-^18^ there appears to be little to choose between BNP and NT-proBNP. The comparisons included factors such as age, gender and renal function. The choice of analyte would therefore be determined by institutional availability, as either would give an accurate reflection of ventricular function. A factor to consider is the differing half-lives; this will influence the timing of sampling in dynamic testing.

The aim of this study was to assess whether NT-proBNP can be used in an unselected population to diagnose exercise-induced myocardial ischaemia.

## Materials and methods

In the study, we enrolled 51 consecutive patients referred for exercise-stress single-photon emission computed tomography (SPECT) myocardial perfusion imaging (MPI) to exclude reversible myocardial ischaemia. The studies were performed at the Nuclear Medicine Department of the Johannesburg and Chris Hani-Baragwanath Hospitals. The study had institutional ethics approval and informed consent was obtained from the subjects prior to enrolment into the study.

The study period was between January and October 2006. The median age of the study population was 58 years (32–82 years) and the male-to-female ratio was 32:19. The exclusion criteria were: asthma or severe chronic obstructive pulmonary disease, beta-blocker use, the inability to reach a target heart rate (these patients were assessed as performing an inadequate stress test and were tested using a dipyridamole stress), and refusal of consent. There were no adverse outcomes during the time of the study.

## SPECT MPI protocol

A two-day 99mTc-methoxyisobutyl isonitrile (Sestamibi) SPECT MPI protocol was used. On the first day the stress test was carried out, with the rest study done on day two. Exercise was performed on a treadmill, using a Bruce protocol; ^99m^Tc sestamibi (740 MBq) was given at peak stress while the subject continued to exercise for one minute after the injection of the radiotracer. The patient was allowed to rest and images were acquired between 30 and 60 minutes after the injection of the radiotracer. Imaging was performed using a double-head rotating field-of-view gamma camera (Infinia Hawkeye, GE Medical Systems) equipped with low-energy, high-resolution, parallel-hole collimators. SPECT images were acquired on a 64 × 64 matrix using the step-and-shoot mode with a total angular range of 180 degrees and 20 seconds per image.

## MPI assessment

Myocardial ischaemia was assessed visually and by means of semi-quantitative scoring of the extent of disease (summed difference score = SDS). This is calculated by subtracting the summed resting score (SRS) from the summed stress score (SSS), using the Xeleris multisync fitted with the QGS/QPS, which uses a 20-segment model to quantify myocardial perfusion on an arbitrary scale. The sensitivity of the SPECT MPI technique in diagnosing ischaemic heart disease is 91% and the specificity is 81%,^19^ compared to exercise-stress ECG with a sensitivity ranging from 37 to 100% and a specificity of 83%.^7^,^20^

Three trained nuclear physicians performed the MPI assessments; they were blinded to the NT-proBNP results. An SDS of more than 4 indicates the presence of myocardial ischaemia. This can be graded: an SDS of 4–8 is mild, 9–12 is moderate and greater than 13 is severe myocardial ischaemia.^21^ A visual inspection of the MPI scan was carried out to assess the perfusion defect when there was any doubt about the diagnosis or the extent of the myocardial ischaemia.

## Blood sampling

In the literature, blood sampling of BNP and NT-proBNP was done within minutes of completing exercise. These studies assessed BNP and NT-proBNP, or only BNP, or only NT-proBNP. 6,7,22-24 Due to the differing half-lives of BNP and NT-proBNP, we performed a limited trial to assess the optimal timing of blood sampling for NT-proBNP and found the ideal time to be 30 minutes after the cessation of exercise, however there is no clear evidence as to the optimal timing of blood sampling.

A resting blood sample was obtained before exercise and the post-stress blood sample was obtained 30 minutes after the cessation of exercise. The sample was allowed to clot and then centrifuged at 3 000 rpm for 10 minutes. The serum was stored at –70°C and analysed in batches.

## NT-proBNP assay

NT-proBNP was measured using kits supplied by Roche Diagnostics, SA. The assay is an electrochemiluminescence immunoassay that uses a sandwich technique. The analytical range is 5–35 000 pg/ml. The reference cut-off value based on the manufacturer’s data is 125 pg/ml. Patients with values below this can exclude cardiac dysfunction with a high level of certainty and levels above 125 pg/ml may indicate cardiac dysfunction, and are associated with an increased risk of cardiac complications (myocardial infarction, heart failure, death). This value was not corrected for age or gender (women tend to have higher values than men and values increase with age). The analytical coefficient of variation for the assay was 1.8–2.7%.

## Statistical analysis

The statistical analysis was done using SAS Software V9.1 (SAS Institute Inc, Cary, NC, USA). The Spearman correlation coefficient was used to calculate correlations, and the Kruskal-Wallis test was used to assess differences in NT-proBNP between the three groups: subjects with no perfusion defects, fixed perfusion defects and inducible perfusion defects. The Wilcoxon test was used to determine differences at rest and post stress for NT-proBNP.

## Results

Of the 51 subjects, 28 had normal perfusion scans (no perfusion defects), seven had fixed perfusion defects (previous infarct, no ischaemia) and 16 subjects had reversible perfusion defects (inducible ischaemia).

**Fig. 1.**
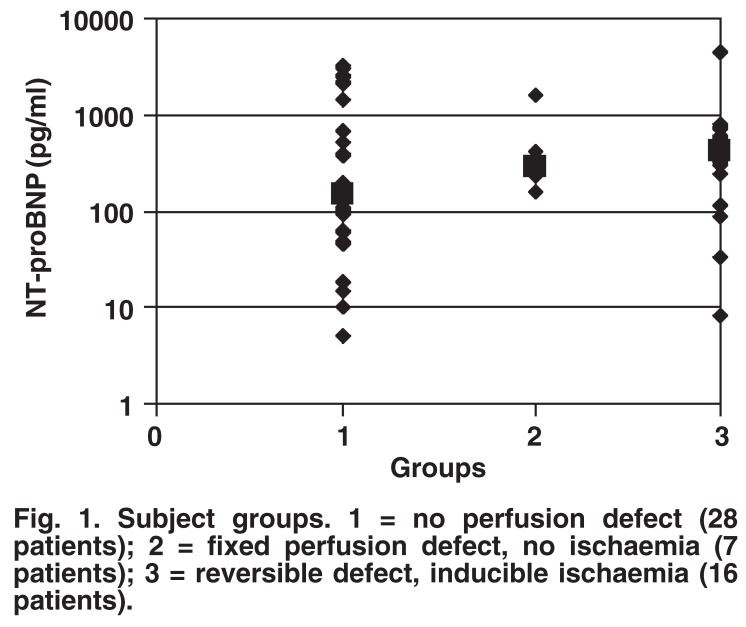
Subject groups. 1 = no perfusion defect (28 patients); 2 = fixed perfusion defect, no ischaemia (7 patients); 3 = reversible defect, inducible ischaemia (16 patients).

The median resting NT-proBNP of all the subjects was 296 pg/ml, with a range of 5–4 463 pg/ml. The highest median resting NT-proBNP result was in the subjects with reversible perfusion defects, followed by those with fixed perfusion defects, and the lowest was seen in the patients with normal MPI scans. These differences were not statistically significant. The characteristics of the study group are summarised in Table 1 and Fig. 1.

**Table 1:** Study Group’s Characteristics

	*Normal*	*Fixed defect*	*Reversible defect*
Number of subjects	28	7	16
Age (years)*	59.5 (32–79)	59 (33–74)	55.5 (36–82)
Male:female	15:13	5:2	12:4
Resting end-diastolic volume (ml)*	86 (34–331)	114 (64–174)	116.5 (64–381)**
Resting NT-proBNP (pg/ml)*	147.5 (5–3165)	281 (157–1594)	416 (8–4463)
Resting ejection fraction (%)*	56 (22–83)	45 (25–61)	38.5 (9–61)***

*Median values (range); **p < 0.05; ***p < 0.01 between the three groups.

**Fig. 2.**
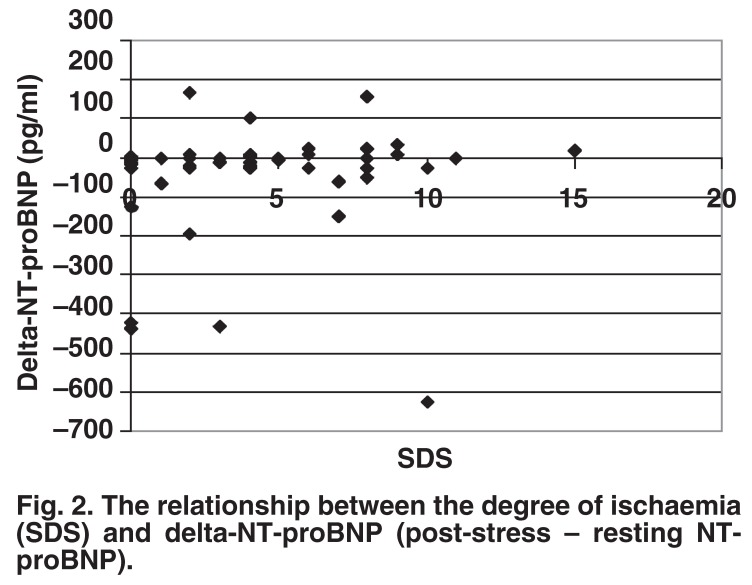
The relationship between the degree of ischaemia (SDS) and delta-NT-proBNP (post-stress – resting NT-proBNP).

**Fig. 3.**
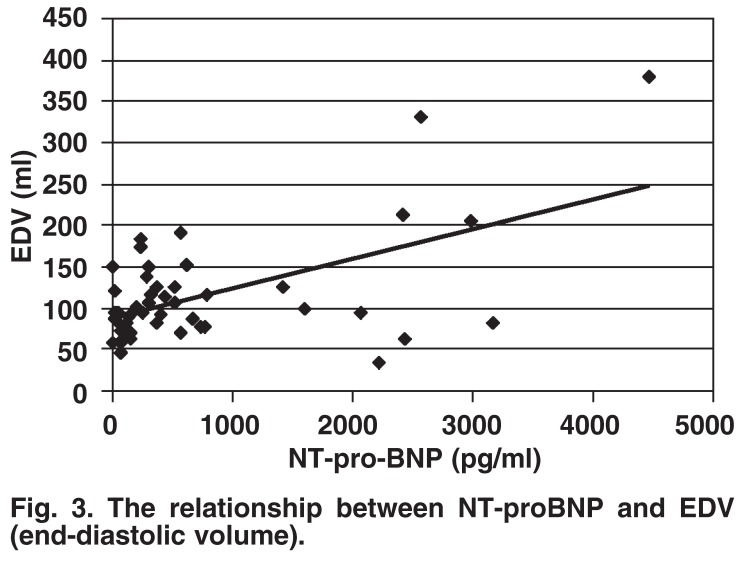
The relationship between NT-proBNP and EDV (end-diastolic volume).

There was no correlation between the index of ischaemia (SDS) and resting, post-effort, or delta-NT-proBNP (post-effort – resting NT-proBNP) (Fig. 2). However, there was a correlation between resting NT-proBNP and ventricular function as reflected by end-diastolic volume (*r* = 0.34, *p* = 0.01) (Fig. 3), and ejection fraction (*r* = –0.43, *p* = 0.0014), as well as endsystolic volume (*r* = 0.38, *p* = 0.006).

## Discussion

The utility of NT-proBNP lies in the fact that it is a sensitive marker of ventricular dysfunction. Myocardial ischaemia, chronic or acute, induces ventricular dysfunction and causes an elevation of NT-proBNP. This elevation can be used as part of risk stratification and prognosis.^5^,^25^,^26^

The relationship between B-type natriuretic peptides and exercise has been extensively studied. Exercise has been shown to increase the secretion of B-type natriuretic peptides in normal individuals, as well as in individuals with myocardial ischaemia. Exercise-induced myocardial ischaemia causes a more significant increase, and in individuals with normal ventricular function it can be diagnostic of myocardial ischaemia. However, there is a marked discrepancy in this regard as to which values to use; some studies find resting values diagnostic,23,24,27-29 and others find the change in values^7^,^8^,^22^,^30^,^31^ or the peak values^32^,^33^ the most important in the diagnosis of myocardial ischaemia.

The change in the secretion of BNP has been shown to improve the diagnostic sensitivity of the exercise-stress ECG. In a selected group of subjects with ejection fractions greater than 50%, the sensitivity of the stress ECG was more than doubled.^7^ In a bigger, non-selected group of subjects, this improvement in sensitivity of the stress ECG when combined with serial monitoring of NT-proBNP was still statistically significant but weak.^33^ This questions the value of serial monitoring of NT-proBNP in the evaluation of exercise-induced myocardial ischaemia in an unselected population.

This study population was unselected and included subjects with ejection fractions less than 45%. In this population there was no relationship between delta-NT-proBNP, resting NT-proBNP or post-effort NT-proBNP and the index of myocardial ischaemia (SDS). The median NT-proBNP was highest in subjects with reversible ischaemia, however there was a large overlap in values.

The secretion of BNP is a dynamic process, which is dependent on mechanical (ventricular stretch) as well as endocrine factors.^34^ Ventricular function is also important; the dysfunctional ventricle can secrete BNP more readily than the normal ventricle. The individual with good LV function is able to perform a more adequate stress test, with a longer duration of exercise, a higher heart rate and higher sympathetic tone, potentially causing a greater stimulus for the release of BNP. These different factors (ventricular function, volume status, sympathetic tone) form a complex interaction when considering the secretion of NT-proBNP and its ability to diagnose inducible myocardial ischaemia.

This is the most likely reason for the differing responses seen in the literature. In subjects with a normal ejection fraction, exercise-induced ischaemia causes a rise in NT-proBNP; in unselected individuals there is no correlation. This lack of correlation is also observed in individuals undergoing pharmacological stress testing.^35^-^37^

Possible limitations of the study are the small number of subjects in the study and the optimal timing of blood sampling for NT-proBNP after exercise. This may have influenced the results.

## Conclusion

No relationship could be demonstrated between exercise-induced myocardial ischaemia and a change in NT-proBNP. This suggests that the serial measurement of NT-proBNP in the routine assessment of exercise-induced myocardial ischaemia is not indicated.
